# A simple exercise to encourage precise suture placement

**DOI:** 10.1308/003588412X13373405385214r

**Published:** 2012-07

**Authors:** RL Storey, MR Gouda, AM Smith

**Affiliations:** Leeds Teaching Hospitals NHS Trust,UK

Surgical simulation is becoming increasingly important due to a reduction in operative exposure during surgical training. We describe a method to encourage precise suture placement using simple equipment that can be practised outside the skills laboratory.

**Figure 1:**
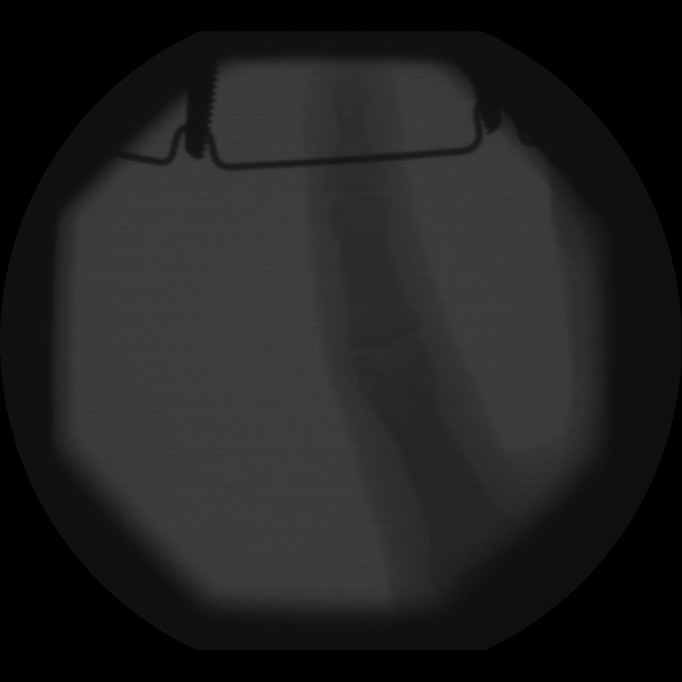
Initial suture placement

**Figure 2:**
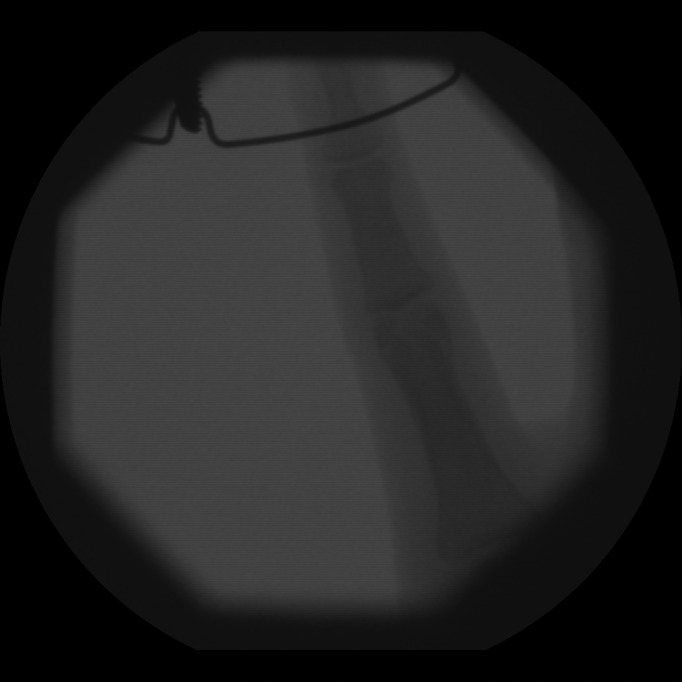
Accurate continuous suture practice

Affix a Post-it® note to a table with its adhesive edge lying to your left. Using an ‘inside to outside, outside to inside’ technique, suture along the free edge of the Post-it® note (Fig 1). Now, pull out the stitch and resuture through the prior made perforations (Fig 2). This simple exercise encourages delicate handling to minimise tissue trauma and a good needle holder technique to facilitate the placement of precise stitches under direct vision.

